# Discovery of HSPG2 (Perlecan) as a Therapeutic Target in Triple Negative Breast Cancer

**DOI:** 10.1038/s41598-019-48993-6

**Published:** 2019-08-28

**Authors:** Stephen Kalscheuer, Vidhi Khanna, Hyunjoon Kim, Sihan Li, Deepali Sachdev, Arthur DeCarlo, Da Yang, Jayanth Panyam

**Affiliations:** 1 Department of Pharmaceutics, University of Minnesota, Minneapolis, MN 55455 USA; 2 Center for Pharmacogenetics, Department of Pharmaceutical Sciences, University of Pittsburgh, Pittsburgh, PA 15261 USA; 3 Department of Medicine, University of Minnesota, Minneapolis, MN 55455 USA; 4 Masonic Cancer Center, University of Minnesota, Minneapolis, MN 55455 USA; 5 Agenta Biotechnologies, Inc., Birmingham, AL 35203 USA; 6 Present Address: Massachusetts Institute of Technology, Cambridge, MA 02139 USA

**Keywords:** Cancer immunotherapy, Target identification

## Abstract

In recent years, there have been significant advances in the treatment of breast cancer resulting in remarkably high survival rates. However, treatment options for metastatic triple negative breast cancer (TNBC) are quite limited due to a lack of identifiable, unique markers. Using a phage display-based whole cell biopanning procedure, we developed two human antibodies that bind to tumor cells with a metastatic TNBC phenotype. Our studies further identified domain 1 of HSPG2 (perlecan) protein as the cognate cell surface antigen bound by the antibody. Immunohistochemistry studies utilizing patient tissue samples revealed significant cell surface expression of HSPG2 in both primary tumors and metastatic lesions. Further, higher HSPG2 expression correlated with poor survival in TNBC. The affinity-matured antibody inhibited the growth of triple negative MDA-MB-231 tumors to a greater extent in nude mice than in NSG mice, pointing to the potential role of natural killer cell-mediated antibody-dependent cell cytotoxicity. This mechanism of action was confirmed through *in vitro* assays using mouse splenocytes and human peripheral blood mononuclear cells (PBMCs). These results suggest that HSPG2 is a promising target in metastatic TNBC and HSPG2-targeted antibodies could represent a potentially novel class of targeted therapeutics for TNBC.

## Introduction

Triple negative breast cancer (TNBC) constitutes 10–15% of all breast cancers^1^. It is characterized by the absence of estrogen receptor, progesterone receptor and human epidermal growth factor receptor 2 (HER2) expression. Patients diagnosed with TNBC have a significantly lower probability of survival^1^. Unfortunately, TNBC patients do not respond to endocrine therapy or other available targeted agents and are typically treated with traditional cytotoxic agents. Further, one-third of these patients progress to advanced disease, and have a life expectancy of less than one year^1^. The significant gap in treatment options for TNBC patients has motivated the search for new therapeutic targets using genomic and proteomic databases^2–4^. However, these efforts have had limited success so far^4,5^.

Phage display is an effective method for generating target-specific antibody or peptide fragments and for potentially identifying new targets^6^. This method utilizes large, diverse libraries of bacteriophage, displaying antibody fragments on their surface, for *in vitro* high-throughput screening against a desired peptide/antigen. Sequential enrichment of antibody fragments that display specificity for the given target eventually leads to a manageable number of candidates that can be tested for specificity. One key advantage of phage display is its potential to identify new targets in their native, physiological form. This can be particularly useful in cases where targets are not known or have been difficult to identify. In the present study, we utilized a whole cell, competition-based phage display procedure to identify high affinity binders to metastatic TNBC cells. Our goal was to identify new target(s) that could be leveraged for therapeutic interventions in TNBC.

An isogenic cell line pair, consisting of human mammary epithelial cells (HMLE) and a Twist-induced metastatic derivative (HMLE-Twist), was used to screen for binders specific to the metastatic TNBC phenotype. The isogenic nature of the cell line pair provided stringent selection of markers relevant to the induction of metastasis. A candidate scFv demonstrating selective binding to metastatic cells was identified, affinity matured and subsequently reformatted to human IgG. Our studies further identified heparan sulfate proteoglycan 2 (HSPG2) as the cognate cell surface antigen bound by the antibody.

HSPG2, also known as perlecan, is a heavily glycosylated protein component of the extra-cellular matrix (ECM) that has been shown to play an important role in tethering and presentation of growth factors to receptors^7^. However, HSPG2 expression in breast cancer has not been examined comprehensively^8–10^. More importantly, expression of HSPG2 in TNBC and its use as a therapeutic target have not been previously explored. We investigated the expression of HSPG2 in human TNBC and the ability of anti-HSPG2 antibodies to specifically target and inhibit tumor growth in a mouse xenograft model. Our results suggest that HSPG2 is a promising therapeutic target in TNBC.

## Results

### Development of TNBC selective Tw1S4_6 scFv and IgG

**Figure 1:**
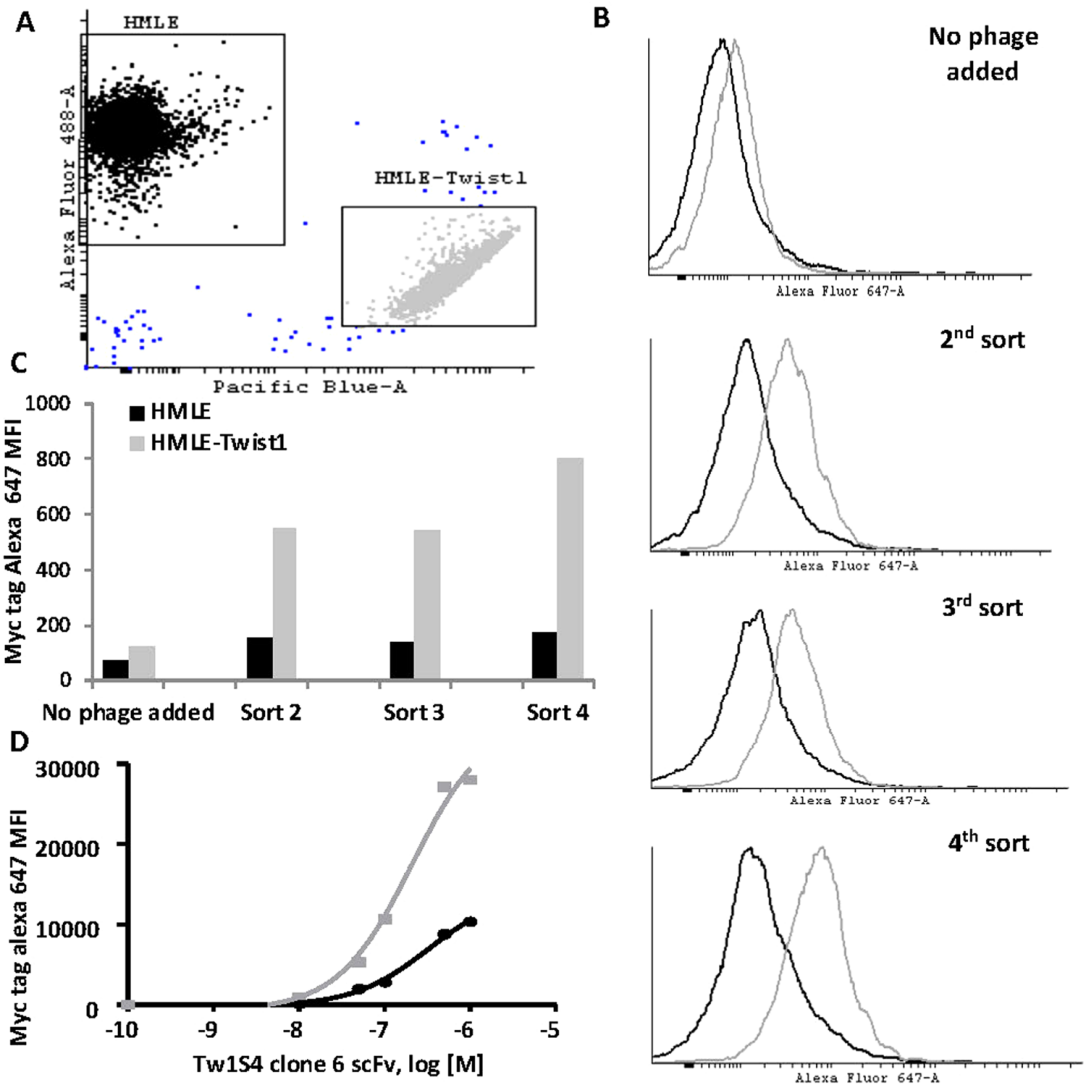
Phage display-based competitive cell panning. HMLE cells were labeled with CFSE and HMLE-Twist1 cells were labeled with Calcein Violet separately, washed, and mixed at a 1:1 ratio. 10^9^ phage from the relevant libraries were added to the cell mixture and incubated with agitation for 30 min at 4 °C. Cells were subsequently washed and labeled with an anti-c-myc secondary AF647 antibody. **(A)** Representative dot plot of phage enrichment experiment. **(B)** HMLE and Twist1 cell populations were analyzed for AF647 fluorescence intensity to determine relative binding of polyclonal phage libraries **(C)** Graphical depiction of data in B. **(D)** Tw1S4 Clone 6 scFv was identified as selective binder to HMLE-Twist1 cells (squares) relative to HMLE (circles), hereafter referred to as Tw1S4_6 scFv.

The Tomlinson scFv phage display library was panned against an isogenic mammary epithelial cell line pair, HMLE (normal) and HMLE-Twist1 (malignant) (characterized in Supplementary Fig. 1), using flow cytometry-based cell sorting (Fig. 1A). A mixing ratio of 100:1 HMLE:Twist1 provided a selective pressure for deriving binders that are selective to Twist1 cells via creation of a cell surface antigen sink provided by excess HMLE cells. A clear distinction in relative binding of sub-libraries to HMLE-Twist1 cells was discernable by the second sub-library (Fig. 1B). This distinction increased to ~8-fold selective binding in the fourth sub-library (Fig. 1C). A monoclonal candidate scFv (designated Tw1S4_6 scFv) was isolated from the fourth sorted sub-library and demonstrated selective binding to Twist1 relative to HMLE cells (Fig. 1D). Reformatting of Tw1S4_6 scFv to human IgG1 was accomplished via PCR amplification of V_H_ and V_L_ and subsequent sub-cloning of the variable domains into pFuse2ss vectors (designated Tw1S4_6) (Supplementary Fig. 2A). Tw1S4_6 retained its selectivity to HMLE-Twist1 cells as determined by flow cytometry (Supplementary Fig. 2B).

### Tw1S4_6 IgG binds to HSPG2/Perlecan Domain 1

**Figure 2:**
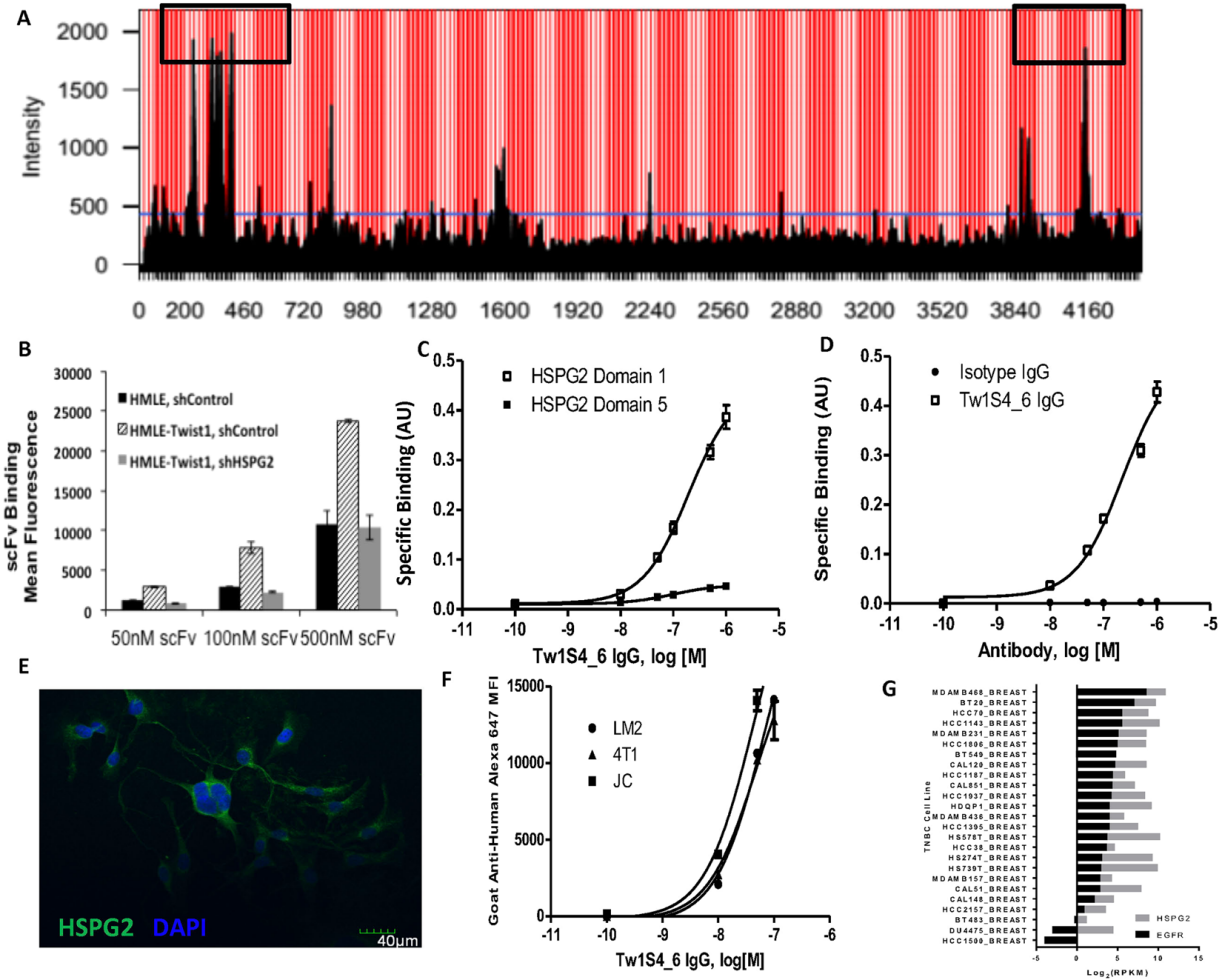
Target deconvolution and Tw1S4_6 IgG reformatting, characterization. **(A)** Linear epitope mapping. The epitope mapping used linear HSPG2 amino acid sequence. Tw1S4_6 binding site was identified in a high-throughput ELISA format in the regions designated by black boxes. **(B)** Assessment of the relative binding of Tw1S4_6 scFv to HMLE-Twist1 cells after HSPG2 knockdown. **(C)** ELISA demonstrating Tw1S4_6 IgG selectively binds HSPG2 domain 1 relative to domain 5. **(D)** Confirmation of Tw1S4_6 selective binding to HSPG2D1 in IgG format. **(E)** Immunofluorescence microscopy of MDA-MB-231-LM2 cells with commercial HSPG2 antibody A7L6. **(F)** Binding titration curves for Tw1S4_6 IgG to metastatic breast cancer cell lines MDA-MB-231-LM2, 4T1, JC Scale bar = 40 μM. **(G)** mRNA expression of HSPG2 and EGFR in TNBC cell lines (Broad Institute Cancel Cell Line Encyclopedia).

To elucidate the target antigen bound by Tw1S4_6, HMLE-Twist1 cell lysates were passed over immobilized Tw1S4_6 scFv. Acrylamide gel resolution of heat denatured scFv-antigen complex revealed a single, high molecular weight antigen co-eluting with Tw1S4_6 scFv (Supplementary Fig. 3A). Tryptic digestion of the excised antigen band followed by MS/MS identified two separate peptides that corresponded to sequences within HSPG2 Supplementary Fig. 3B), providing initial evidence for HSPG2 as the binding partner for Tw1S4_6. HSPG2 knockdown in HMLE-Twist cells (Supplementary Fig. 3C) resulted in reduced binding of Tw1S4_6 scFv (Fig. 2B), confirming the binding partner for Tw1S4_6 scFv was HSPG2. Linear epitope mapping was employed to further elucidate the binding region of Tw1S4_6. Several pockets of concentrated binding were observed at N-terminal residues <500, and C-terminal residues >3800 (Fig. 2A) i.e., the first and fifth domains of HSPG2, respectively^11^. Recombinant HSPG2 domain 1 (HSPG2D1) was expressed and purified as previously reported^12^, with HSPG2D5 being available commercially. ELISA based evaluation demonstrated specific binding of Tw1S4_6 to HSPG2D1 over HSPG2D5 (Fig. 2C). Another ELISA experiment comparing Tw1S4_6 IgG with isotype IgG binding to HSPG2D1 demonstrated specific binding of Tw1S4_6 to HSPG2D1 (Fig. 2D). Increasing concentrations of free HSPG2D1, but not HSPG2D5, were able compete off Tw1S4_6 IgG binding to MDA-MB-231-LM2 cells, providing confirmation that the target epitope on these cells was indeed HSPG2 domain 1 (Supplementary Fig. 3D). We also investigated the effect of heparinase pre-treatment on Tw1S4_6 binding to MDA-MB-231-LM2 cells. As shown in Supplementary Fig. 3E, heparinase treatment reduced the binding of the antibody significantly. This suggests that glycosaminoglycan residues (present in HSPG2D1 and to some extent HSPG2D5) are important components of the epitope recognized by the antibody.

### HSPG2 is expressed *in vitro* in human and mouse TNBC cell lines

Our initial extension to relevant *in vitro* models of cancer focused on cell lines bearing predisposition to invasion and metastasis. HSPG2 expression in MDA-MB-231-LM2 cells was confirmed via immunofluorescence microscopy using a commercially available HSPG2 antibody (clone A7L6) (Fig. 2E). Following that, binding curves for Tw1S4_6 were generated for three metastatic breast cancer cell lines: breast to lung metastatic MDA-MB-231-LM2 (human), 4T1 (mouse) and JC (mouse) (Fig. 2F). We also investigated HSPG2 gene expression in several TNBC cell lines using the Broad Institute’s Cancel Cell Line Encyclopedia (www.portals.broadinstitute.org/ccle) and found HSPG2 expression in 22 out of 25 cell lines (Fig. 2G). In most cases, HSPG2 expression was higher than EGFR, a commonly investigated therapeutic target for TNBC^5,13,14^.

### HSPG2 is expressed in human tumors and correlates with poor patient survival

**Figure 3:**
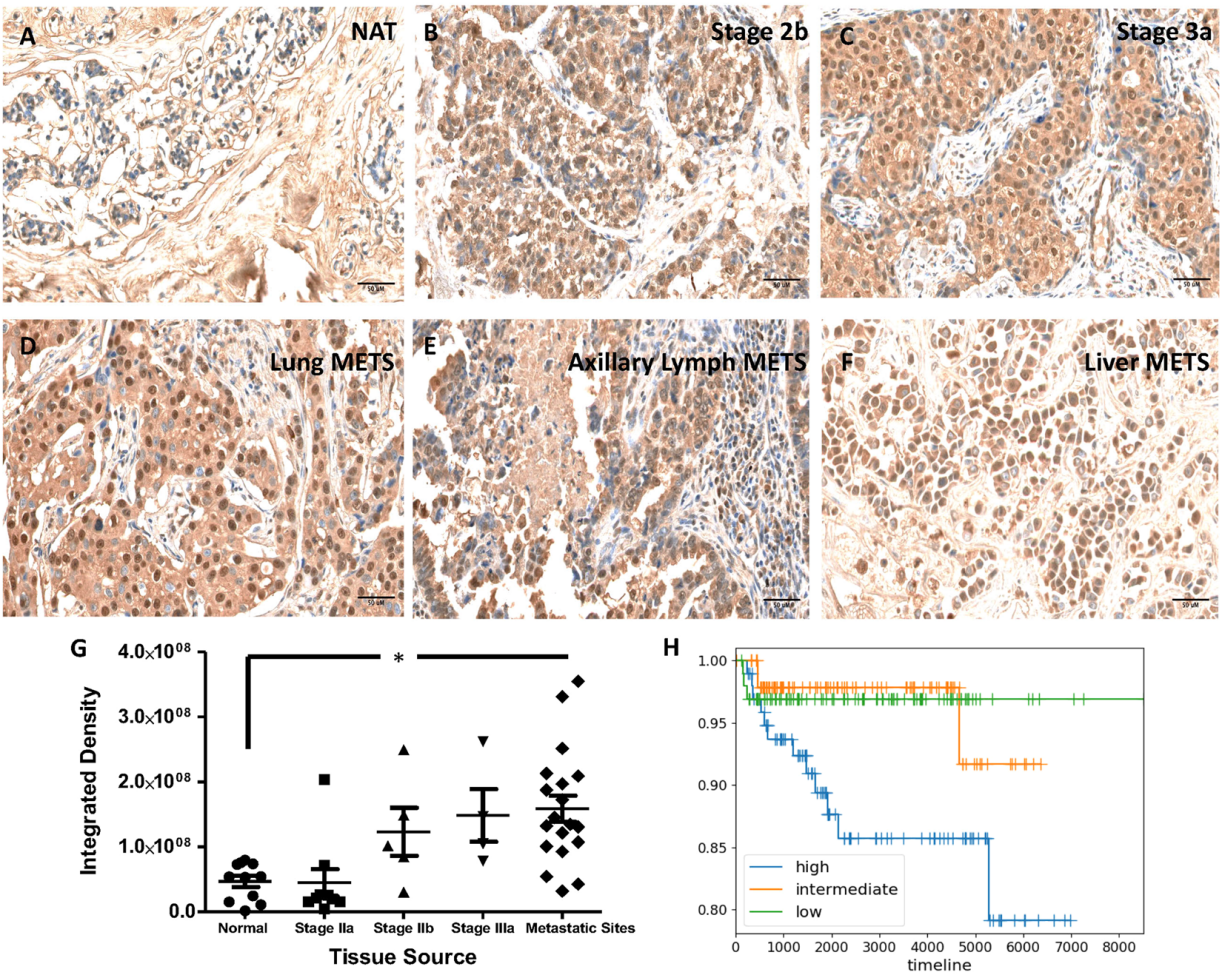
Immunohistochemistry for HSPG2 expression in human tissue microarrays T088b, MET961 (US Biomax) **(A**–**F)** Tw1S4_6 was used to stain a breast cancer tissue microarray. The staining pattern of HSPG2 changes from interstitial to predominantly cellular with advancing stage. Images were acquired at 200x magnification using an automated slide scanner. Pictures shown are snippets captured from the original images at 20X digital zoom. **(G)** HSPG2 expression is observed at increasing intensity with advancing tumor stages, the highest being tumor metastatic sites (*P < 0.05; one-way ANOVA with Kruskal Wallis post-test) **(H)** Survival Analysis based on HSPG2 Expression. All patient and HSPG2 expression data was obtained from METABRIC. For patients with TNBC, high HSPG2 expression correlates with significantly poorer survival (P < 0.01, multi-group log-rank test).

We next examined HSPG2 expression in human tumors with Tw1S4_6. Normal adjacent breast tissue (NAT) was lightly stained in the extra-cellular matrix compartment (Fig. 3A), whereas stage 2 and 3 tissues revealed a strong cellular and nuclear staining (Fig. 3B,C). Importantly, intense staining of malignant cells was observed in breast cancer-derived liver, lung and lymphatic metastases (Fig. 3D–F), pointing to the continued expression of HSPG2 in metastatic cells. Staining with a control Isotype IgG is shown in Supplementary Fig. 4A–C. Quantification of staining revealed that HSPG2 expression increased with advancing tumor stage, with the highest expression being observed at metastatic sites (Fig. 3G).

To determine whether there was an association between HSPG2 expression and disease prognosis, we conducted an analysis using the METABRIC database [PMID: 27161491]. In the case of all breast cancer subtypes, differential expression of HSPG2 had no correlation with patient survival (P > 0.05) (Supplementary Fig. 4D). When the pre-stratified analysis was narrowed to patients with TNBC, we observed a significant decrease in survival with higher HSPG2 expression in TNBC (P < 0.05) (Fig. 3H). Univariate cox regression analysis to assess the overall association between HSPG2 expression and patient survival revealed a hazard ratio of 7.945, indicating that higher HSPG2 expression is associated with a significantly poorer survival (P < 0.01) (Supplementary Table 1).

### Affinity Maturation of Tw1S4_6 Antibody

**Figure 4:**
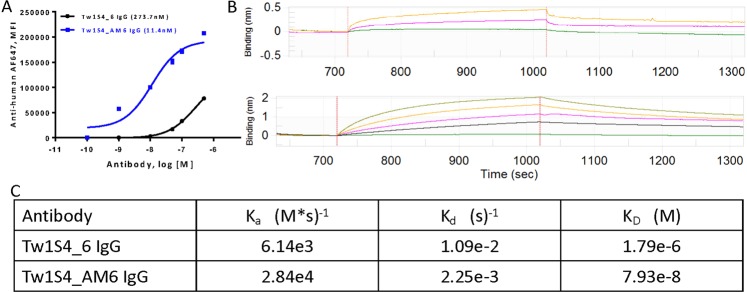
Evaluation of affinity matured antibody Tw1S4_AM6. **(A)** Flow cytometry-based evaluation of K_D_ value in MDA-MB-231-LM2 cells, calculated K_D_ values mentioned in legend **(B)** Biolayer inferometry curves for Tw1S4_6 (Top Panel) at 100 nM (green), 500 nM (pink), 1 µM (yellow) and Tw1S4_AM6 (Bottom Panel) at 10 nM (green), 50 nM (black), 200 nM (pink), 400 nM (dark green). **(C)** K_a,_ K_d_, and K_D_ values obtained from curves generated in (**B**).

While Tw1S4_6 demonstrated good selectivity for HSPG2, its low affinity (K_D_~125–275 nM) was a limitation. Thus, prior to evaluating the therapeutic potential of anti-HPSG2 antibody, the binding affinity of Tw1S4_6 was improved through mutagenesis of key residues within complementarity determining regions (CDRs). This led to the development of an affinity matured IgG termed ‘Tw1S4_AM6’. The apparent K_D_ for Tw1S4_AM6, estimated from flow cytometry binding curves to MDA-MB-231-LM2 cells, was ~10 nM - a greater than 10-fold improvement in binding affinity compared to Tw1S4_6 (Fig. 4A).

An important caveat with flow cytometry-based titration curves is the potential for avidity affects contributing to binding, resulting in an ‘apparent K_D_’^15^. Thus, biolayer interferometry was used as a means of assessing the true, analytically relevant ‘intrinsic K_D_’ (Fig. 4B). Surprisingly, the parent antibody Tw1S4_6 returned a modest K_D_ value at ~1.8 µM (Fig. 4C). Affinity matured Tw1S4_AM6 demonstrated substantial monovalent affinity improvement to HSPG2D1, having a K_D_ value of 80 nM (Fig. 4C). While we did not repeat the full linear epitope mapping studies with Tw1S4_AM6, the biolayer interferometry studies confirmed binding of Tw1S4_AM6 to HSPG2D1. We also confirmed that Tw1S4_AM6 stained both primary TNBC tumors and breast cancer-derived metastasis similar to Tw1S4_6 (Supplementary Fig. 5).

### Tw1S4 antibodies are effective in inhibiting tumor growth *in vivo*

**Figure 5:**
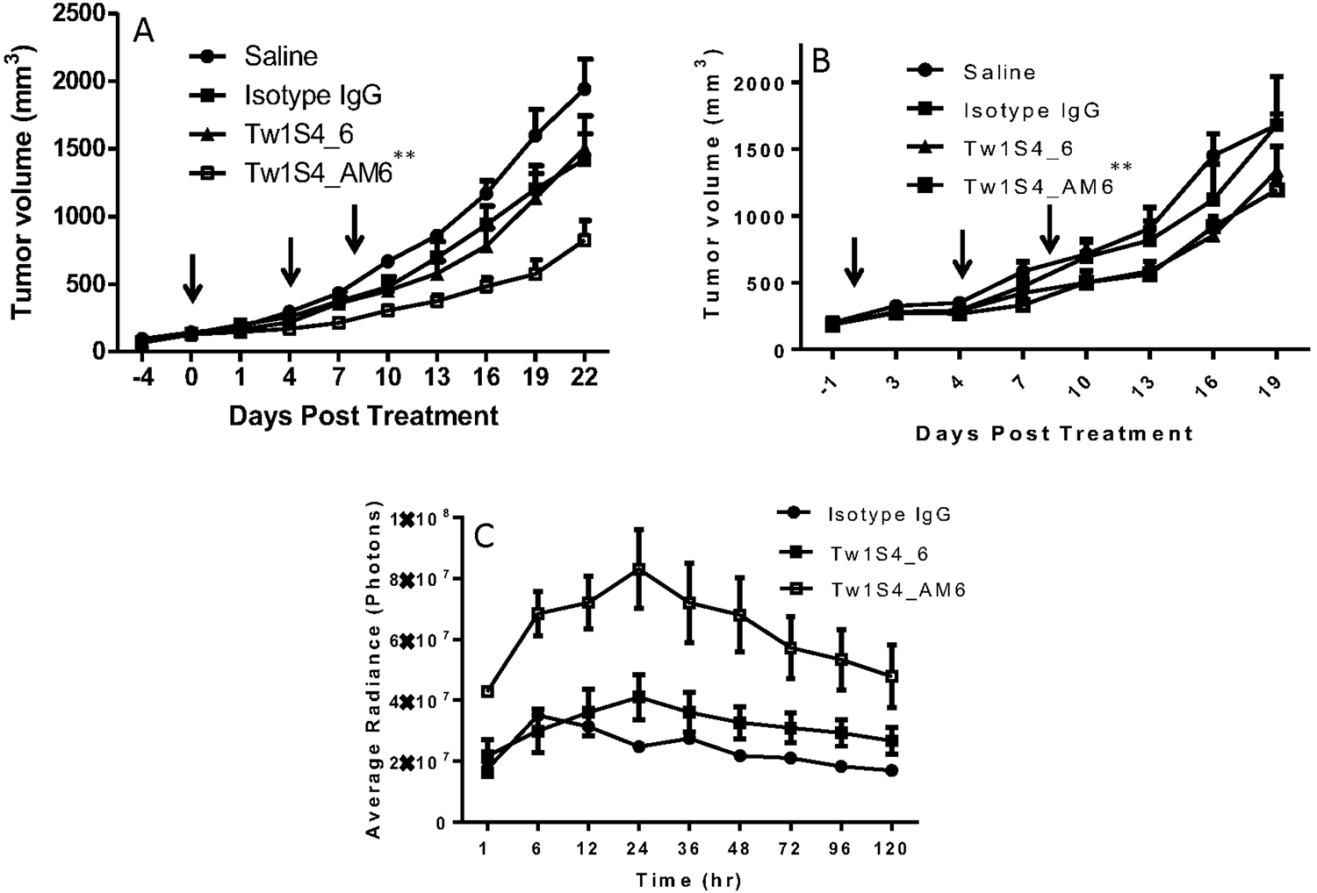
*In vivo* efficacy and biodistribution studies for Tw1S4 antibodies. MDA-MB-231-LM2 tumors were grafted subcutaneously. For efficacy studies (A and B) dosing begun when the tumor volumes were greater than 100 mm^3^. All the antibodies were dosed at 5 mg/kg, 3 doses, every 96 hours (indicated by arrows). **(A)** Efficacy study in athymic nude mice. Tw1S4_AM6 showed significant tumor inhibition (**P < 0.01, two-way ANOVA with multiple comparisons, statistical significance is based on comparison between isotype IgG and Tw1S4_AM6 on the last day of the study) **(B)** Efficacy study in NSG mice. Tw1S4_AM6 resulted in significant but blunted efficacy (**P < 0.01, two-way ANOVA with multiple comparisons, statistical significance is based on comparison between isotype IgG and Tw1S4_AM6 on the last day of the study). **(C)** Quantified fluorescence values in tumor. Imaging was done when tumors reached 300 mm^3^. 100 µg of antibody was injected per mouse. Imaging was carried out at the time points indicated in the figure. Tw1S4_AM6 accumulated between 2–4-fold higher concentrations in the tumor (***P < 0.001, two-way ANOVA with multiple comparisons, statistical significance is based on comparison between isotype IgG and Tw1S4_AM6 at 24 hours).

Human IgGs are capable of engaging with murine FcγR expressed on various immune effector cells^16^. MDA-MB-231-LM2 tumor grafted in Balb/c athymic nude mice was used as an *in vivo* model capable of facilitating ADCC when using human IgGs. Tw1S4_6 IgG produced marginal tumor growth inhibition relative to the saline treated group (Fig. 5A). Tw1S4_AM6, on the other hand, significantly inhibited tumor growth, resulting in a mean tumor volume of 500 mm^3^ on day 22, relative to saline and isotype control groups, which had mean tumor volumes >1,500 mm^3^.

To evaluate the specific contribution of immune based effector cells in the mechanism of anti-HSPG2 antibodies, we used the severely immunocompromised NSG mouse model^17^. An identical graft model in NSG mice led to a statistically significant reduction in tumor volumes for Tw1S4_6 and Tw1S4_AM6 (Fig. 5B). The reduction in tumor volumes however, was blunted relative to that in the Balb/c nude model, suggesting a significant contribution of immune based effector cells to the efficacy of Tw1S4_AM6. Further experiments are required to understand what drives the residual efficacy observed in NSG mice for both the antibodies.

To investigate the tumor distribution of the two antibodies, an imaging study was carried out in tumor-bearing mice with fluorescently labeled antibodies. Following IV administration, fluorescence was visible in the liver at one hour. Starting at 12 hours, significant accumulation of the antibodies could be observed in the tumor. Quantitative analysis of fluorescence in the tumors revealed a 2–4 fold higher accumulation of Tw1S4_AM6 versus the isotype IgG control (P < 0.05 at 6 and 72–120 hours, p < 0.001 at 12–48 hours) (Fig. 5C and Supplementary Fig. 6A,B), similar to reports testing other tumor-targeting antibodies^18,19^. Tw1S4_6 on the other hand showed a modest, statistically not significant, improvement in accumulation (1.5- to 2-fold, P > 0.05). The T_max_ in tumor for both Tw1S4_6 and Tw1S4_AM6 was 24 hours. On the contrary, T_max_ in the liver was the first measured time-point i.e. 1 hour (Supplementary Fig. 6B) and there were no statistically significant differences in the liver distribution of the three antibodies (P > 0.05).

### Tw1S4_AM6 mediates ADCC *in vitro* with mouse splenocytes and human PBMCs

**Figure 6:**
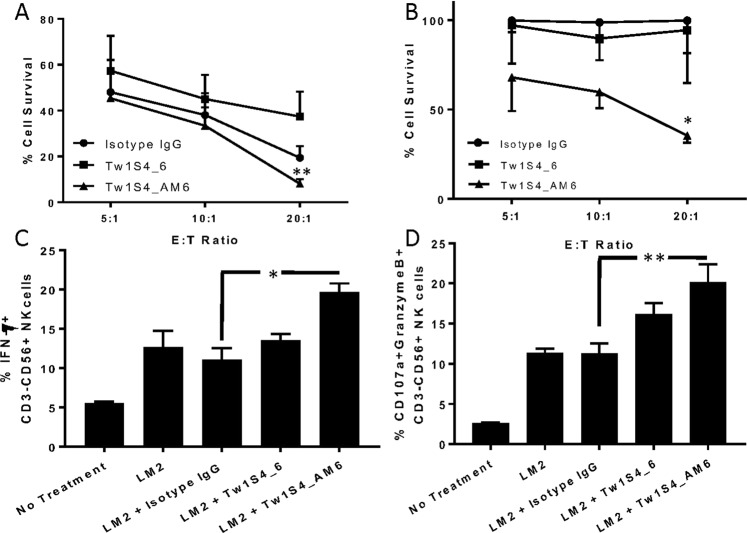
*In vitro* ADCC assays with Tw1S4 antibodies. **(A)** ADCC assay with mouse splenocytes showed improved cytotoxicity with Tw1S4_AM6 (**P < 0.01, two way ANOVA with Tukey’s multiple comparison tests, statistical significance is based off comparison between Tw1S4_6 and Tw1S4_AM6 at E:T 20:1) **(B)** ADCC assay with human PBMCs from donor 1 showed significantly higher cytotoxicity with Tw1S4_AM6 (*P < 0.05, two way ANOVA with Tukey’s multiple comparison tests, statistical significance is based off comparison between Isotype IgG and Tw1S4_AM6 at E:T 20:1) **(C)** and **(D)** NK cell degranulation assays with human PBMCs from donor 1 (*P < 0.05, **P < 0.01, one way ANOVA with Tukey’s multiple comparison tests).

The observed differences in efficacy in athymic nude versus NSG mouse models suggested the possible involvement of immune based effector cells in mediating the anticancer efficacy of Tw1S4 antibodies, in particular antibody dependent cellular cytotoxicity (ADCC). We carried out *in vitro* ADCC assays with mouse splenocytes^15^. Tw1S4_AM6 induced ADCC resulting in cell survival of 8%, as opposed to 33% with Tw1S4_6 and 19% with isotype IgG at an effector-to-target (E:T) ratio of 20:1 (Fig. 6A). A similar trend was observed at lower E:T ratios of 10:1 and 5:1. The inclusion of IL2 in the assay resulted in high non-specific cell kill. However, previous reports suggested that human antibodies do not induce cell lysis with mouse effector cells unless stimulated^15^. It was not surprising that Tw1S4_6 showed no ADCC *in vitro* considering that antibody affinity has been shown to be a critical parameter in determining ADCC outcome^15^. Another possible mechanism exerted by antibodies is complement dependent cytotoxicity (CDC). However, we did not observe any cytotoxicity *in vitro* with either mouse or human serum (Supplementary Fig. 8).

In order to test the translational potential of the Tw1S4 antibodies, we also tested *in vitro* ADCC and NK cell degranulation with healthy human PBMCs from two donors. At an E:T ratio of 20:1, we observed that treatment with Tw1S4_AM6 resulted in a significantly higher tumor cell kill (35% viability as opposed to 99% and 94% in case of Isotype IgG and Tw1S4_6 antibodies, respectively) (Fig. 6B and Supplementary Fig. 6A), with similar trends at lower ratios. We also observed an increase in the fraction of CD3^−^/CD56^+^ cells secreting interferon-γ (Fig. 6C), a cytokine responsible for T cell activation (11% for Isotype IgG v/s 19% for Tw1S4_AM6) and those positive for CD107a (Fig. 6D), a degranulation marker (11% for Isotype IgG v/s 20% for Tw1S4_AM6). A similar trend was observed in studies with PBMCs from a second donor (Supplementary Fig. 7B,C), however the overall cytotoxicity observed was lower in the case of donor 2.

## Discussion

Breast cancer is the most common type of cancer in women, accounting for 30% of all diagnosed cases in the United States^20^. Most breast cancer subtypes have a good prognosis when the cancer is diagnosed early, because of the availability of targeted therapies^21^. These include tamoxifen, anastrozole, exemestane, and letrozole for ER+/PR+ sub-types and trastuzumab for HER2+ sub-type^21^. Unfortunately, not many druggable targets have been identified in TNBC.

Phenotype-based screening is well suited to identify functional candidate antibodies that bind to a relevant phenotype, capturing the target antigen as it would exist in its physiologically relevant form. This provides a direct route to antibody based drug or diagnostic reagents that could be readily translated into therapies^22^. Given that prior knowledge of a potential target is not needed, this approach is also highly amenable to novel biomarker discovery via target deconvolution.

Two antibodies, spanning an order of magnitude in apparent K_D_ towards cell surface HSPG2, were developed from phage display-based whole cell phenotype screening. HSPGs are ubiquitous constituents of extra-cellular matrix (ECM) and vascular basement membranes. Functional roles of HSPG2 are diverse, and include participation in the scaffolding function of ECM, along with collagens, laminins and fibronectin^23–25^. HSPG2 is the primary HSPG within normal tissue ECM and vascular basement membranes, and has been previously observed to be affiliated with the surface of malignant cells of colon carcinoma^26^, Kaposi’s sarcoma^27^ and melanoma^28^, as well as in the tumor stroma of various cancers^29^. Downregulation of HSPG2 expression using antisense in colon carcinoma cells resulted in decreased tumor growth and angiogenesis^26^. A similar decrease in proliferation occurred in melanoma, Kaposi’s sarcoma cell lines and more recently in prostate cancer cells *in vitro* and *in vivo*, following HSPG2 downregulation using antisense^27,30,31^. Additional studies are needed to understand the mechanisms behind the dramatic shift in HSPG2 localization from the stromal compartment in normal adjacent tissue to the surface of malignant cells and to the nuclei in invasive carcinoma as well as the significant increase in HSPG2 expression with tumor stage. Other studies have reported similar nuclear localization of perlecan and other HSPGs^32,33^. While the functions of cell surface/nuclear HSPGs are still incompletely understood, it has been suggested that they may affect gene transcription and may have possible roles in cell proliferation^32^. Interestingly, it has been previously reported that an increase in matrix metalloproteinase enzymes in the tumor stroma leads to a reduction of HSPG2 expression in the ECM, thus favoring cell dispersion and invasiveness^34^.

In our studies, we observed a strong correlation between HSPG2 expression and survival of TNBC patients. In pancreatic adenocarcinoma, progressive loss of stromal HSPG2 expression was shown to correlate with disease progression^35^. Similarly, in glioblastoma, high HSPG2 expression in the tumor correlated with poor relapse-free survival^36^. In our analysis, this correlation was observed specifically in the case of TNBC patients and not in other subtypes in our studies.

Given the success of Trastuzumab against HER2 + breast cancer^37^, there is considerable interest in developing similar antibody-based therapies for TNBC. The *in vivo* efficacy observed with Tw1S4_AM6 as a single agent treatment points to a potential, exciting new antibody-based therapeutic approach in TNBC. ADCC is an immune effector mechanism mediated primarily by NK cells. Most antibodies (IgG1 subtype) are able to simultaneously engage target-expressing cells through the Fab domain and NK cells through the Fc domain^38^. This leads to activation of NK cells, followed by a release of cytotoxic proteins such as perforin and granzyme, resulting in the death of target cells^39^. Antibodies such as Trastuzumab and Cetuximab, which are primarily known for inhibition of signaling pathways, also induce ADCC^40,41^. The ability of Tw1S4_AM6 to mediate cell kill via ADCC opens up several options to enhance its efficacy through specific mutations in the Fc portion^42^ or the reduction of core fucosylations to improve ADCC^43^. Another option is to combine antibodies with NK cell stimulating agents^44,45^. Such improvements in efficacy could be particularly beneficial to patients with the phenylalanine isoform of FcγRIIIa^46^. The *in vivo* results also suggest that further refinement of the dosing regimen may be required to achieve durable responses, considering that cetuximab (anti-EGFR antibody) is dosed at 10–40 mg/kg for at least five doses in rodent xenograft models^47^. Additionally, it would be interesting to evaluate Tw1S4_AM6 in other cancer sub-types such as glioblastoma^36^ and prostate cancer^31^ that were previously demonstrated as having high HSPG2 expression.

In summary, the cell phenotype-based screening approach outlined here enabled the identification of HSPG2 as a novel therapeutic target in TNBC. This led to the development of Tw1S4_AM6, an anti-HSPG2 human IgG antibody, which mediates anti-cancer efficacy through ADCC. Identification of HSPG2 as a unique tumor cell antigen and the development of a fully human antibody demonstrating therapeutic efficacy represent a potential new point of focus in TNBC.

## Methods

All experiments were performed in accordance with relevant guidelines and regulations. All experimental protocols using animals were reviewed and approved by University of Minnesota Institutional Animal Care and Use Committee (IACUC) and experiments were performed accordingly. Human PBMCs were isolated from blood of healthy adult volunteers. Informed consent was obtained from all subjects and the blood collection was approved by University of Minnesota Institutional Review Board (IRB).

### Cell culture conditions

HMLE isogenic cell lines were a generous gift from the lab of Dr. Robert Weinberg. HMLE is an immortalized human mammary epithelial cell line. HMLE-Twist1 cells are derived by inducing the expression of Twist transcription factor in HMLE cells as described by Mani *et al*.^48^. HMLE-Twist1 cells have been characterized as an EMT transformed cell line^48^. HMLE cells and HMLE-Twist1 cells were cultured in MEGM media (Lonza). MDA-MB-231-LM2 cells were derived from lung metastasis developed upon intravenous injection of the parental MDA-MB-231 cells in mice as described by Minn *et al*.^49^. and were obtained from Dr. Joan Massague of the Howard Hughes Medical Institute, Memorial Sloan Kettering Cancer Center (New York, NY, USA). Cells were cultured in MEM supplemented with 10% v/v FBS and antibiotics. JC and 4T1 cell lines were cultured in RPMI supplemented with 10% v/v FBS and antibiotic. All cell culture materials were obtained from Thermo Fisher Scientific (Waltham, MA) unless otherwise specified.

### Phage display biopanning procedure

The Tomlinson phage display library was used for the biopanning procedure. Propagation and purification of bacteriophage were performed as per supplier’s protocol (Source BioScience, Santa Fe Springs, CA). An *in vitro* competition-based panning procedure that utilized an isogenic cell line pair (HMLE and HMLE-Twist1) was developed. HMLE is an hTERT immortalized primary breast epithelial cell line^50^. Overexpression of EMT-inducing transcription factor Twist1 in HMLE cells results in global changes in gene expression resulting in increased invasiveness^51^ and cancer stem cell (CSC)-like behavior^48^. The presence of E-cadherin and the absence of vimentin in HMLE and their inverse expression profile in HMLE-Twist1 was used to confirm the phenotypic switch in the two cell lines (Supplementary Fig. 1A). In addition, HMLE-Twist1 cells are less sensitive to chemotherapy relative to HMLE (Supplementary Fig. 1B) and exhibit CD44^hi^/CD24^low^ phenotype (Supplementary Fig. 1C), ascribed to the cancer stem cells (CSC). For competitive cell panning, two fluorescent cell viability dyes, Calcein AM450 and CFSE, were used to discriminate HMLE, from a sub-population of HMLE-Twist1 (Fig. 1A). HMLE cells were labeled with 10 μM CFSE at 10^6^ cells/mL and mixed at 100:1 ratio with Calcein AM-450 labeled HMLE-Twist1 cells. Dye labeling was performed according to manufacturer’s protocol (eBioscience, San Diego, CA).

For the first pan, the naive phage library was added to the mixed cell suspension in DPBS. The target HMLE-Twist1 cells were then sorted from the mixed population using BD FACS Aria cell sorter. Phage bound to the sorted HMLE Twist1 cells were eluted in pH 2 glycine buffer, and propagated in TG1 bacteria to generate sub-libraries, designated Tw1_S1 through Tw1_S4. After four cell-sorting experiments, the sub-libraries were evaluated for enrichment of phage selective for Twist1 cells. The same two-color discrimination scheme was employed. After labeling, 1 * 10^9^ PFU of phage from each sub-library were incubated with a 1:1 cell suspension HMLE and HMLE-Twist1 cells. Analysis of phage bound to cells was determined with an anti-myc-epitope (9E10, Abcam, Cambridge, UK), followed by anti-mouse dylight 650 secondary (Sigma Aldrich, St. Louis, MO). Tw1S4_6 scFv was isolated from Tw1_S4, which demonstrated selective binding to HMLE-Twist1 relative to HMLE cells (Fig. 1D). Phagemid clone analysis for both polyclonal and monoclonal populations was done via flow cytometry staining. Further details can be found in Supplementary Information.

### Coarse linear epitope mapping against HSPG2

Linear 20-mer peptides corresponding to human HSPG2 having a 16-residue overlap with adjacent sequence, were synthesized in an array format by Pepscan (Netherlands) using proprietary methods. Tw1S4_6 IgG as well as a commercially available human IgG isotype antibody (Sigma) were each tested for binding to the 20-mer peptides in an ELISA format by Pepscan.

### Flow cytometry-based determination of antibody binding

MDA-MB-231-LM2 cells were trypsinized and aliquoted to 10^5^ cells in PBS (200 µL). Cells were incubated with a range of antibody concentrations for 60 minutes at 4 °C on a rotating shaker. Flow cytometry buffer used was PBS, 0.5% w/v BSA, 2 mM EDTA. Following two wash steps, a goat anti-human Dylight 647 conjugate antibody was used to determine the extent of antibody binding to cells. A BD LSR2 was used to analyze cells for fluorescence. Non-linear regression analysis was performed using the geometric MFIs from antibody binding titrations, using GraphPad Prism software. The 50% value from regression analysis is presented as the apparent K_D._ This procedure was used for all cell lines tested for Tw1S4_6 or Tw1S4_AM6 binding.

### ELISA

HSPG2 domains 1 and 5 were coated onto ELISA plates for 2 hours at room temperature, using a concentration of 10 µg/mL. Following three PBS washes, plates were blocked with blocking buffer (PBS, 2% v/v BSA) for 2 hours at room temperature. Tw1S4_6 IgG, diluted in blocking buffer at the indicated concentrations, was plated in triplicate and incubated overnight at 4 °C. The plates were then washed and incubated with 1:20,000 Protein-L HRP (Genscript) for 2 hours at room temperature. Three washes in PBS followed by incubation with TMB substrate (Sigma) for 15 minutes were performed. The peroxide solution was quenched with stop solution (Sigma), and signal intensity was quantified on a UV/Vis 96-well plate reader (BioTek Instruments, Vermont, USA) by measuring absorbance at 450 nm. Log concentration - response plots were generated in GraphPad Prism software. Nonlinear regression analysis was employed to generate fitted curves to the data.

### Tissue microarray immunohistochemistry

Human tissue microarrays (Catalog Numbers T088b, MET961, BR1002a) were purchased from USBiomax (Rockville,MD). Tw1S4_6 or Tw1S4_AM6 antibodies were used for evaluation of perlecan expression. Standard immunohistochemistry was performed by the Comparative Pathology Shared Resource at the University of Minnesota. Images were captured by the University of Minnesota Imaging Centre through brightfield slide scanning at 200X magnification. Images shown are snippets captured at 20X digital zoom. Quantification of HSPG2 staining was done using ImageJ software (NIH).

### HSPG2 expression and survival analysis

HSPG2 and EGFR mRNA expression data as obtained from the Broad Institute’s Cancer Cell Line Encyclopedia. The breast cancer cell lines were classified into sub-types based on Lehman *et al*.^52^ and Jian *et al*.^53^.

To investigate the association between HSPG2 mRNA expression and patient survival in breast cancer, we performed a log-rank test for pre-stratified patient groups by expression level. For each cancer type, patients were divided into three groups based on HSPG2 expression from high (first third), intermediate (second third) to low (last third) (n = 1085). Considering the grouping methods are arbitrary, univariate cox regression analysis was applied to assess the overall association between HSPG2 expression and patient survival for each cancer type.

To investigate the association between mRNA HSPG2 expression and patient survival with triple-negative (ER-, PR-, Her2-) breast cancer (TNBC), log-rank test for pre-stratified patient groups was used. For analysis, patients (n = 298) were divided into three groups based on HSPG2 expression from high (first third), intermediate (second third), low (last third). Considering the grouping method is somewhat arbitrary, univariate cox regression analysis was also applied to assess the overall association between HSPG2 expression and patient survival. Considering that other factors, including patient age at diagnosis, tumor grade, size, stage and number of positive lymph nodes, would also influence the survival, multivariate cox regression was also used (n = 223). All profiled patient samples and HSPG2 expression are from METABRIC.

### Biolayer interferometry-based determination of antibody binding kinetics

We used biolayer interferometry (BLItz, Fortebio) to determine antibody binding kinetics specifically with purified HSPG2 domain 1. Ni-NTA biosensors were hydrated for 10 minutes in phosphate buffered saline. Following hydration, a 30 second baseline was established, followed by a 10-minute loading of the biosensor with 10 µg/mL HSPG2 domain 1 protein. The biosensor was then placed in PBS to establish baseline for 30 seconds. A two-step antibody association – dissociation cycle of 5 minutes each was then used to determine the kinetic association (k_a_) and dissociation (k_d_) rate constants. The antibodies were used at concentrations varying between 100 nM to 1μM for Tw1S4_6 and 10 nM to 400 nM for Tw1S4_AM6, in PBS. The equilibrium dissociation constant (K_D_) was calculated as K_D_ = k_d_/k_a_.

### Tumor growth inhibition studies

MDA-MB-231-LM2 tumors were grafted in Balb/c homozygous nude mice (Charles River Labs or Jackson Labs) or Balb/c NSG mice. One million viable cells resuspended in 1:1 saline:matrigel (Corning, NY) in 100 μL were grafted subcutaneously into the fourth mamillary fat pad. Once tumor volumes reached 100 mm^3^, three doses (5 mg/kg) of the antibodies were administered through tail vein injection once every 96 hrs. Isotype human IgG and saline were used as controls (n = 7 for nude mice, and n = 4–6 for NSG mice). Tumor volumes were measured every third day with an electronic caliper. Tumor volumes were calculated from the ellipsoid sphere equation V = (L^2^ * W)/2, L being the longer measurement. Two Way ANOVA, with multiple comparison post-tests, was used to determine the statistical significance of the data.

### Kinetics of antibody tumor accumulation

All experimental protocols using animals were reviewed and approved by University of Minnesota Institutional Animal Care and Use Committee (IACUC) and experiments were performed accordingly. Mice bearing MDA-MB-231-LM2 tumors (~300 mm^3^) were injected with 100 µg of labeled isotype IgG Control, Tw1S4_6 or Tw1S4_AM6 (n = 3). Antibodies were labeled with Cy 7 maleimide (Click Chemistry Tools, Arizona) using immunothiolation to introduce reactive thiols onto the antibody. Mice were imaged using the IVIS Spectrum *In Vivo* Imaging System (University of Minnesota Imaging Centre) at various time intervals over 120 hours using an excitation/emission filter of 750/775 nm. Data was acquired and analyzed using living image software. Statistical data was obtained using Two Way ANOVA, with multiple comparison post-tests.

### Antibody-dependent cellular cytotoxicity (ADCC) assays with human PBMCs and mouse splenocytes

Human PBMCs (effector cells) from healthy donors were purified using Ficoll-Paque density gradient media (GE Healthcare), incubated overnight at 37 °C in RPMI (10% v/v FBS, 1% v/v P/S) and used for the assay the next day. Target cells (MDA-MB-231-LM2) were labeled with 8 µM CFSE (Biolegend, CA, USA) for 20 minutes at room temperature, followed by two washes. CFSE labeled target cells were then incubated with 100 nM of the relevant antibodies in suspension at 4 °C for 1 hour, washed once and then used for the assay. Effector and target cells were incubated together in 96 well plates, at the specified ratios overnight at 37 °C. The next day, plates were centrifuged at 1200 RPM for 5 minutes and 50 µL of supernatant was collected and cell cytotoxicity was measured using LDH assay kit (Thermo Fisher Scientific, CA, USA). The remaining media was aspirated, followed by addition of 100 µL PBS. The plates were read using a plate reader at Ex/Em 485/528 nm to determine relative cell viability.

Mouse splenocytes were obtained from the spleen of Balb/c homozygous nude mice^15^. The cells were stimulated for 48 hours with 500 units/mL IL2 (R&D Systems, MN, USA), after which they were washed, counted and used for ADCC assay as described above.

### Human PBMC degranulation assay

Human PBMCs (5 × 10^5^/well) were seeded in a 24-well cell culture plate. To achieve sub-optimal activation of PBMCs, polyinosinic–polycytidylic acid (poly I:C) (10 µg/ml) was added to the media. After 18 hr incubation, MDA-MB-231-LM2 cells (2.5 × 10^5^/well) were added to the PBMCs. Subsequently, isotype IgG control, Tw1S4_6 or Tw1S4_AM6 antibodies (200 nM) were added to the media and anti-CD107a antibody was added directly into the media. After 1 hr, brefeldin A solution (Biolegend) was added to the media. Cells were collected after 3 hr and stained with anti-CD3 and anti-CD56 antibodies. Intracellular staining of interferon gamma (IFN-ɣ) and granzyme B were conducted according to the manufacturer’s protocol (Foxp3/Transcription Factor Staining Buffer Kit, Tonbo bioscience). Stained cells were analyzed by flow cytometry (LSRFortessa H0081, BD bioscience).

## Supplementary information

Supplementary methods and results
