# Supplementary material for: Discovery of HSPG2 (Perlecan) as a Therapeutic Target in Triple Negative Breast Cancer
Source: Sci Rep. 2019 Aug 28;9:12492. doi: 10.1038/s41598-019-48993-6 (PMC6713791; doi:10.1038/s41598-019-48993-6)
Supplement: Supplementary file 1 — Supplementary methods and results [file 41598_2019_48993_MOESM1_ESM.docx]

**Discovery of HSPG2 (Perlecan) as a Therapeutic Target in Triple Negative Breast Cancer**

Stephen Kalscheuer^1#^, Vidhi Khanna^1#^, Hyunjoon Kim^1^, Sihan Li^2^, Deepali Sachdev^3,4^, Arthur DeCarlo^5^, Da Yang^2^, Jayanth Panyam^1,4*^

^1^Department of Pharmaceutics, ^3^Medicine, ^4^Masonic Cancer Center, University of Minnesota, Minneapolis, MN, USA 55455

^2^Center for Pharmacogenetics, Department of Pharmaceutical Sciences, University of Pittsburgh, Pittsburgh, PA, USA 15261

^5^Agenta Biotechnologies, Inc., Birmingham, AL 35203

# Both authors contributed equally

**Supplementary Information**

**Supplementary Methods**

**Soluble scFv production**

Tw1S4_6 scFv in phagemid vector was isolated from a single TG1 colony following the fourth round of competitive flow sorting. For soluble scFv production, 1 µg of phagemid DNA was digested with Nco1 and Not1 (New England Biolabs). The scFv sequence was purified following 1% agarose gel electrophoresis using the Quick gel extraction kit (Qiagen). pET22b(+) was digested with Nco1 and Not1. Ligation of the phagemid derived scFv sequence to pET22b(+) was performed using T4 DNA ligase (New England Biolabs) according to manufacturer‘s protocol. A molar ratio of 1:3 vector to insert was used for ligation. The ligated plasmid was transfected into BL21 (DE3) chemically competent cells (New England Biolabs). DNA sequencing was used to confirm successful sub-cloning. Soluble scFv production was induced by the addition of IPTG to a final concentration of 1 mM to log-phase culture of the transformed bacteria, and incubated for 3-4 hours at 30°C. Bacteria were pelleted at 4000 rpm for 30 minutes. The pellet was resuspended in B-PER reagent (Thermo Fisher) to extract the periplasmic fraction containing soluble scFv. This fraction was passed over nickel-NTA agarose resin (5 PRIME), followed by wash and elution using an imidazole gradient (40 mM imidazole for washing, 250 mM for elution). Eluted fractions were concentrated and buffer-exchanged to DPBS using Amicon Ultra centrifugal concentrators (10 kDa cut off) (Millipore, Burlington, MA).

To assess relative binding of soluble phage to cells, scFvs were incubated with indicated cell lines in flow buffer (DPBS, 0.5% w/v BSA, 2 mM EDTA) for 1 hour. Following washes, An anti- 6x His tag dylight 647-conjugated monoclonal antibody recognizing the scFv (AbCAM) was used to assess binding.

**Cognate antigen immunoprecipitation and MS/MS identification**

Tw1S4_6 scFv (300 µg), produced in pET vector, bearing a C-terminal His tag, was immobilized on nickel agarose. Gravity flow chromatography cartridges were used to contain the sample. 1*10^7^ HMLE-Twist1 cells were lysed in 3 mL RIPA buffer. Following centrifugation to clear the insoluble lysate debris, the total cell lysate was passed over scFv-agarose beads, which were subsequently washed four times using 20 mM imidazole in DPBS, pH 7.5, followed by elution of scFv – antigen complex with 250 mM imidazole in DPBS, pH 7.5. The eluent was concentrated using a 10 kDa molecular weight cut-off Amicon ultra-filtration dialysis units. The concentrated sample was prepared for acrylamide gel electrophoresis in Laemmli sample buffer, and boiled for 10 minutes to denature the scFv – antigen complex. Sample was resolved on a 4-15% gradient acrylamide gel, and stained with Coomassie blue to identify scFv and immunoprecipitated antigen. A band of >250kD molecular weight was excised from the acrylamide gel with a sterile scalpel and placed in a micro-centrifuge tube. An in-gel tryptic digestion kit (Pierce) was used according to the manufacturer’s protocol to prepare the excised protein band for MS/MS analysis.

**Database searching**-- Charge state deconvolution and deisotoping were not performed. All MS/MS samples were analyzed using Sequest (Thermo Fisher Scientific, San Jose, CA, USA; version 27, rev. 12). Sequest was set up to search the rs_human_041912_cRAP database (unknown version, 33986 entries) assuming the digestion enzyme trypsin. Sequest was searched with a fragment ion mass tolerance of 0.80 Da and a parent ion tolerance of 1.00 Da. Iodoacetamide derivative of cysteine was specified in Sequest as a fixed modification. Oxidation of methionine was specified in Sequest as a variable modification.

**Criteria for protein identification**-- Scaffold (version Scaffold_4.2.1, Proteome Software Inc., Portland, OR) was used to validate MS/MS based peptide and protein identifications. Peptide identifications were accepted if they could be established at greater than 95.0% probability by the Peptide Prophet algorithm ^1^. Protein identifications were accepted if they could be established at greater than 99.0% probability and contained at least 2 identified peptides. Protein probabilities were assigned by the Protein Prophet algorithm ^1^. Proteins that contained similar peptides and could not be differentiated based on MS/MS analysis alone were grouped to satisfy the principles of parsimony.

**Heparinase treatment of cells**

Recombinant heparinase was purchased from Sigma Aldrich. Cells were trypsinized, washed, and resuspended in DPBS containing 25 mM Tris. Cell suspensions were incubated with 0.5 mU/mL heparinase for 30 min at 37°C with shaking. Cells were washed three times in DPBS prior to antibody staining. Stained cells were analyzed by flow cytometry. The commercial antibodies used in this study were as follows:

A mouse IgM monoclonal specific to heparan sulfate (Amsbio, clone F58-10E4), along with an anti-mouse IgM-APC dye conjugate secondary (Biolegend, Cat# 406509) was used as positive control, to confirm Heparinase activity against target cell lines.

A biotinylated anti-human HSPG2 antibody (Neomarkers, clone A7L6) recognizing a non-glycosylated HSPG2 epitope, along with streptavidin-APC secondary (Biolegend).

**HSPG2 Knockdown in HMLE-Twist**

For stable knockdown of HSPG2 (Perlecan) in the HMLE-Twist1 cell line, lentiviral particles expressing shRNA targeting HSPG2, along with lentiviral particles expressing a scramble control shRNA, were purchased from Santa cruz biotechnology. Viral transduction was performed according to the manufacturer protocol. Stable transformants were selected with 1µg/mL Puromycin for 5 days, and expanded for subsequent experiments. Confirmation of HSPG2 knockdown was determined by flow cytometry.

**scFv reformatting to human IgG1**

Reformatting of Tw1S4_6 scFv to human IgG1 was accomplished via PCR amplification of V_H_ and V_L_ and subsequent sub-cloning of the variable domains into pFuse2ss vectors. The vector system consists of separate constructs expressing constant domains of heavy and light chains. Following sequence verification, the full-length heavy and light chain constructs representing humanized Tw1S4_6 IgG were co-transfected into Expi293 cells. Subsequent affinity-based purification yielded a pure IgG product as determined by PAGE analysis (Supplementary Figure 2A). Flow cytometry studies confirmed that the selective binding to HMLE-Twist1 cells was maintained in the IgG format (Supplementary Figure 2B).

Vectors encoding constant regions of the heavy chain human IgG1 (pFUSE2ss-CHIg-hG1) and light chain κ (pFUSE2ss-CLIg-hk) were purchased from InvivoGen. PCR Primers were designed to amplify the corresponding variable region from Tw1S4_6 scFv, with appended restriction enzyme sites introduced into the primer, according to the manufacturers recommendations. Primers were as follows:

Vl_R_BsiWI: TAAACGTACGTTTGATTTCCACCTTGGT

Vl_F EcoR1: TAAGCAGAAGGCAACGGACATCCAGATGACC

Vh_R_nhe1: AAGCGCTAGTCGCTCGAGACGGTGACCAGGGT

Vh_F_EcoR1: TAAGCAGAATTTCGAGGTGCAGCTGTTGGAGTC

PCR of the corresponding variable domain was performed with Phusion HF DNA polymerase (NEB) and the following thermal cycling protocol:

For V_H_: 98C for 30sec, followed by 35 cycles of [98C – 10sec, 67C – 15sec, 72C – 15sec].

For V_L_: 98C for 30sec, followed by 35 cycles of [98C – 10sec, 65C – 15sec, 72C – 15sec]

PCR amplicons were resolved on a 1% agarose gel, excised and purified using QiaQuick gel extraction kit according to manufacturer protocol (Qiagen). Restriction enzyme (RE) digestion of V_L_ and pFUSE2ss-CLIg-hk was performed with EcoR1 and BsiWI. RE digestion of V_H_ and pFUSE2ss-CHIg-hG1 was performed with EcoR1 and Nhe1. Variable region fragments were again gel purified following RE digestion. RE digested pFUSE2ss vectors were treated with CIP phosphatase (NEB) according to manufacturer protocol and purified from the enzyme reaction with GET Clean DNA plasmid columns (G Biosciences). A molar ratio of 1:3 vector to insert was used for ligation with T4 DNA ligase. The ligation reactions were transformed into NovaBlue *E. coli* strain (Merck Millipore). Proper variable domain insertion into pFUSE2ss vectors was confirmed by sequencing using primer Antibody vector seq: TGCTTGCTCAACTCTACGTC. Detection of Tw1S4_6 IgG binding to cell lines was determined by flow cytometry using a goat anti-human IgG dylight 647 antibody. Flow cytometric analysis was carried out on a BD LSR2 cytometer.

**Expression and purification of HSPG2 Domain 1 and 5**

HSPG2 domain 1 construct was previously reported ^2^. DNA sequence encoding the first 247 amino acids of human HSPG2 was cloned into pcDNA 3.3+ vector bearing a C terminal His tag. The plasmid was used to express domain 1 in suspension HEK cells. The protein was purified using HisPur Ni-NTA Chromatography Cartridges (Thermo Scientific, Illinois, USA). HSPG2 domain 5 was purchased (Santa Cruz Biotechnology).

**Immunofluorescence microscopy**

Cells were plated onto 8-well CC2 chamber slides (Lab Tek), and allowed to adhere prior to fixation with 3% formaldehyde for 10 minutes. Cells were subsequently permeabilized with 0.5% v/v Triton-X 100. Following a 1-hour blocking step in 5% w/v BSA in PBS, cells were stained with dye conjugate antibodies for 1 hour in blocking buffer. Antibodies used in fluorescence microscopy experiments were: PE anti-human E-cadherin (clone 67A4, Biolegend), PE anti-human EpCAM (clone 1B7, Biolegend), Efluor 660 anti-human vimentin (clone V9, eBioscience), Biotinylated anti-human perlecan antibody (clone A7L6, Neomarkers) and streptavidin dylight 488 (Biolegend), or streptavidin APC depending on the other fluorophores in the antibody panel. All the antibodies were used at 1:250 dilution. Following staining, cells were mounted in ProLong Gold anti-fade mounting medium (Life technologies) and imaged within 24 hours. Image acquisition was performed on an Olympus Fluoview FV1000 BX2 upright confocal microscope.

**scFv affinity maturation**

The scFv affinity maturation procedure for Tw1S4_6 was performed as described previously in detail^3^. Briefly, affinity maturation was accomplished via a targeted mutagenesis approach in which 7 amino acids comprising CDR3 of the light chain were mutagenized with a primer containing NNB degenerate codons. The procedure involves two sequential steps. The initial step employs the mutagenic primer, which hybridizes to CDR3 of V_L_, along with LMB3 primer to amplify the full scFv gene and introduce directed mutagenesis. The second PCR employs the same LMB3 primer, as well as a forward primer that contains the remaining C terminal scFv residues, which were lost during the first PCR amplification.

The primers used were as follows:

1. LMB3 primer: CAGGAAACAGCTATGAC
2. V_L_ CDR3 mutagenic primer: CCCTCCGCCGAACACCCAACCNNBNNBNNBNNBNNBNNBNNBCTGGCAGTAATAATCAGCCTC

Vent (exo -) DNA polymerase with deficient 3’ – 5’ proofreading activity was used for mutagenesis (NE Biolabs). Mutagenic PCR thermal cycling conditions were: 94°C for 5 minutes followed by 30 cycles of: 42°C for 1 minute, 72°C for 2 minutes. The mutagenized scFv template was gel purified on a 1% agarose gel. Gel extraction was accomplished with QIAquick PCR purification kit (Qiagen) according to the manufacturer-supplied protocol. Whole cell phenotype screening was utilized to screen the affinity matured library, similar to that used for the development of Tw1S4_6. Candidate affinity matured scFvs were subsequently reformatted to human IgG1. Antibodies were produced and purified in a manner similar to that described above using the Expi293 transfection system and Protein A columns.

**CDC Assays with Mouse and Human Serum**

Target cells (MDA-MB-231-LM2) were incubated with varying concentrations of the relevant antibodies in suspension at 4˚C for 1 hour, washed once and then used for the assay. Target cells were incubated in serum-free media containing 10% normal mouse serum (Sigma-Aldrich, Missouri, USA) or 50% normal human serum (Sigma-Aldrich, Missouri, USA) depending on the assay, in 96 well plates overnight at 37˚C. The next day, 50 µL of supernatant was used for LDH assay (Thermo Fisher Scientific, CA, USA) and the remaining media was aspirated, followed by an MTS assay (Promega, Wisconsin, USA).

**
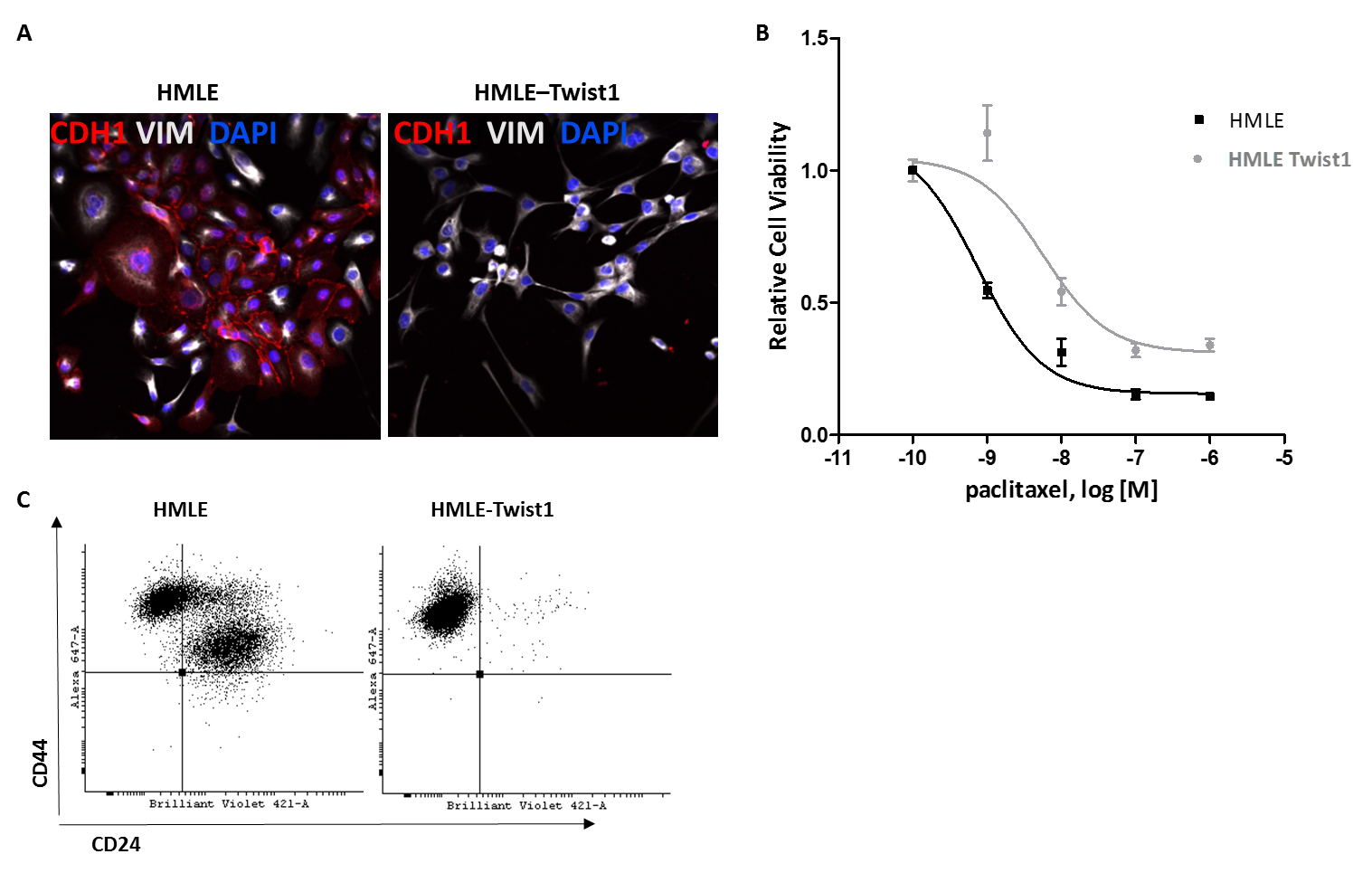
**

**Supplementary Figure 1:** EMT Characteristics of isogenic HMLE cell lines. **(A)** Immunofluorescence staining of HMLE and HMLE-Twist1 cells for canonical epithelial and mesenchymal protein markers E-Cadherin (CDH1) and Vimentin (VIM), respectively. **(B)** Dose-response curve showing differential sensitivity of HMLE and HMLE-Twist1 cells to chemotherapeutic agent paclitaxel. **(C)** Cell surface cancer stem cell immunophenotyping demonstrates HMLE-Twist1 is predominately CD44^+^/CD24^low^ (right panel), relative to HMLE cells which have a mixed phenotype.

**
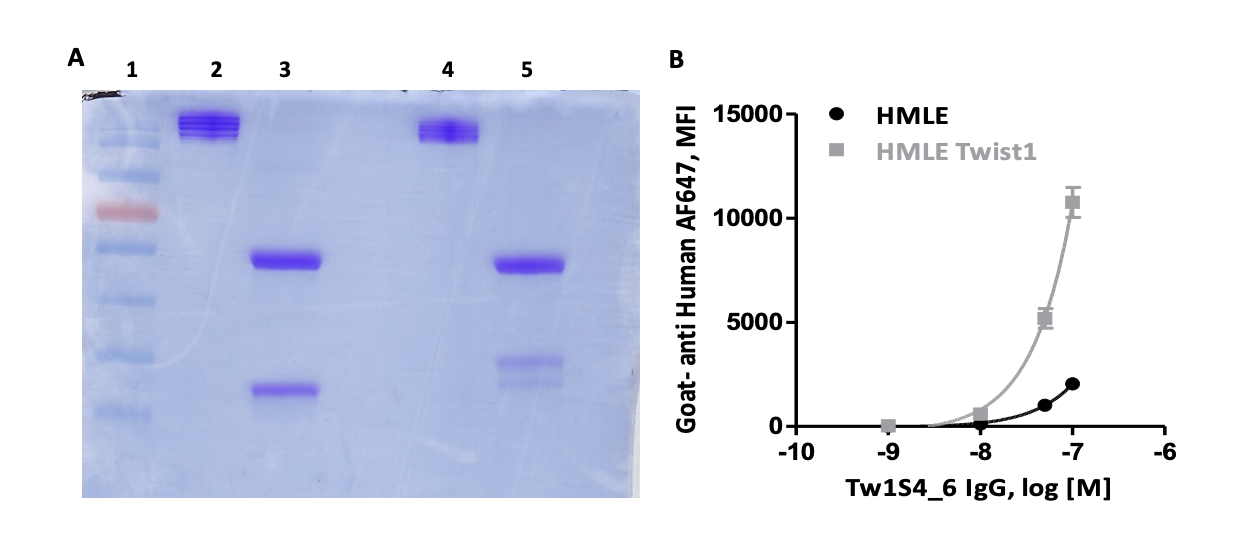
Supplementary Figure 2:** Reformatting scFv to full-length human IgG **(A)** SDS-PAGE based characterization of Tw1S4_6 IgG (Lane 1 – molecular weight ladder, lanes 2 & 3 – Non-reduced and reduced respectively), Tw1S4_AM6 IgG (Lanes 4 & 5 – Non-reduced and reduced respectively) **(B)** Tw1S4_6 IgG retains selectivity to HMLE Twist1 as analyzed by flow cytometry


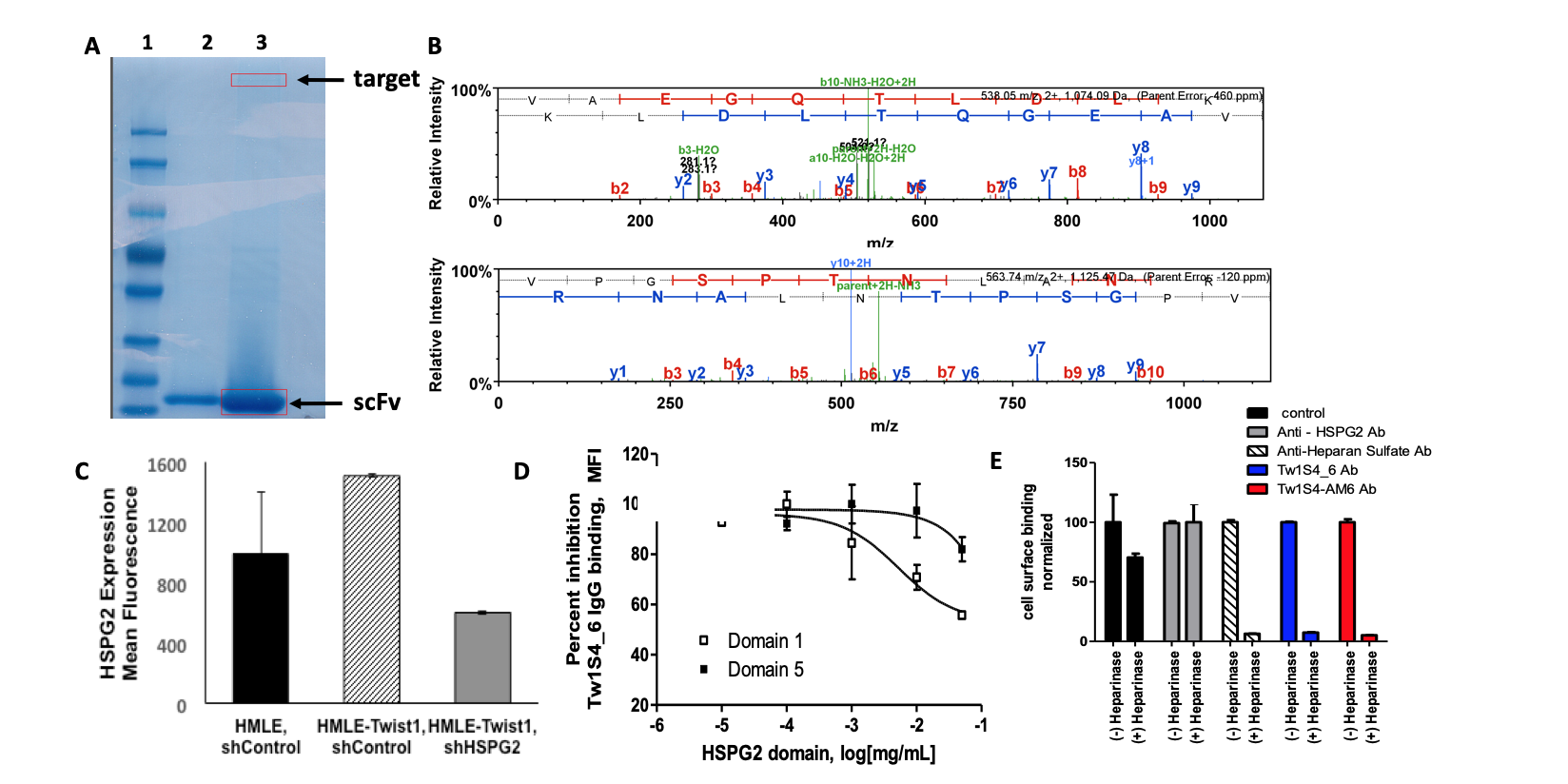


**Supplementary Figure 3:** Tw1S4_6 scFv target deconvolution **(A)** Coomassie staining of acrylamide gel resolved antigen pull down. Tw1S4_6 scFv was immobilized on IMAC resin. Solubilized HMLE-Twist1 cell lysate was applied to Tw1S4_6 scFv and washed extensively in PBS containing 20 mM imidazole. Lane 1 is a molecular weight ladder. Lane 2 represents an aliquot of final wash fraction. Lane 3 represents the elution fraction. **(B)** In-gel tryptic digestion was performed prior to MS/MS. The fragmentation pattern of the two tryptic-digest peptides from target band in (A) confirm HSPG2 as cognate target of Tw1S4_6 scFv. **(C)** Confirmation of HSPG2 knockdown in HMLE-Twist cells using commercial anti-HSPG2 antibody. Note that the absolute MFIs are significantly lower for the commercial anti-HSPG2 antibody relative to Tw1S4_6 ScFv in Figure 2(B). **(D)** Competitive inhibition of Tw1S4_6 IgG binding to LM2 cells with increasing concentrations of soluble HSPG2 domain 1 proteoglycan or domain 5 proteoglycan (E) Cells were evaluated for antibody binding following recombinant heparinase treatment. Control (black bars) represent isotype human IgG staining negative control. An antibody recognizing a non-glycosylated HSPG2 epitope (grey bars) is not influenced by heparinase cell treatment. An antibody specific to heparan sulfate (striped bars) was used as a positive control to confirm effectiveness of heparinase treatment. Tw1S4_6 (blue bars) and Tw1S4_AM6 (red bars) binding is dependent on the presence of cell surface heparan sulfate.


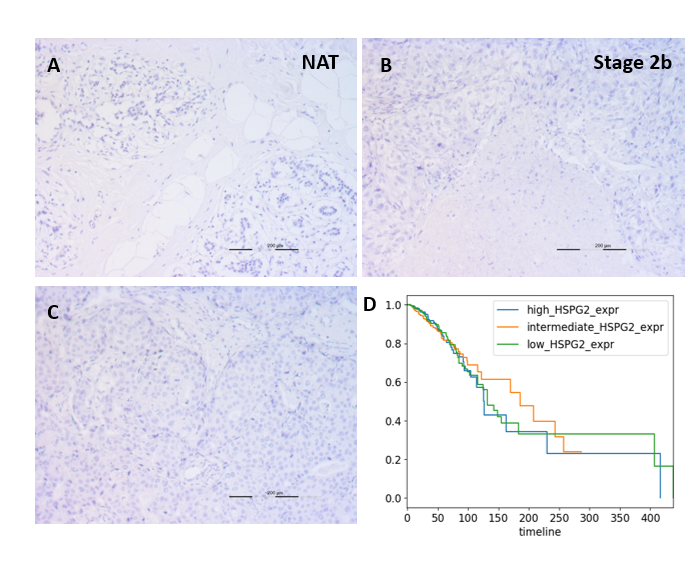


**Supplementary Figure 4:** **(A-C)** Control staining with Isotype IgG expression on human breast cancer tissue microarrays used in Figure 4. **(D)** Survival Analysis based on HSPG2 Expression. All patient and HSPG2 expression data was obtained from METABRIC. For patients with breast cancer, HSPG2 expression does not correlate with survival (P>0.05, multi-group log-rank test).


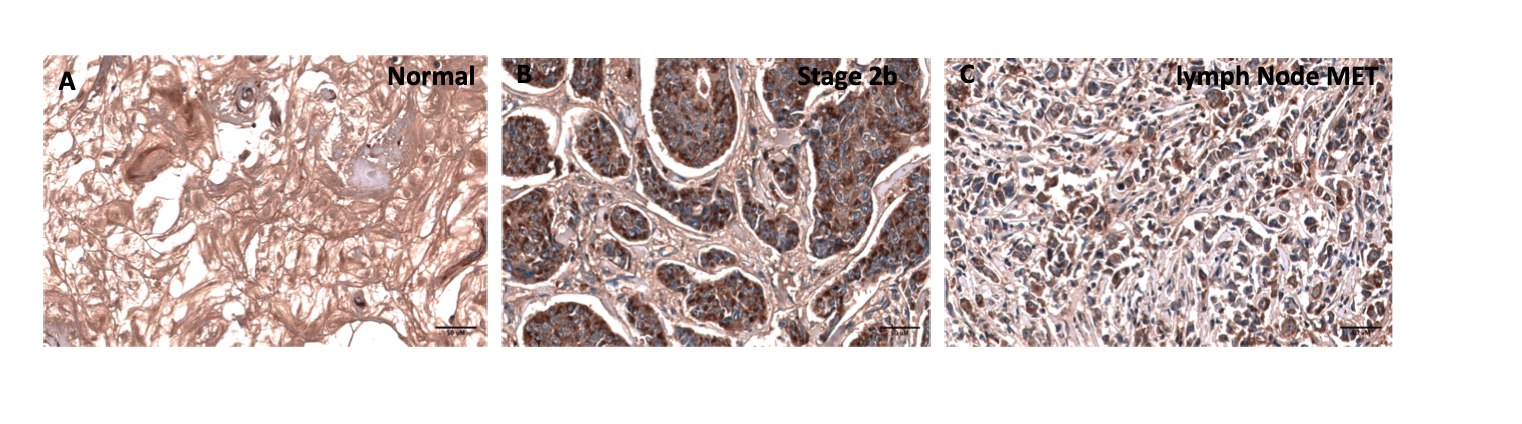
**Supplementary Figure 5**: Immunohistochemistry for HSPG2 expression in human tissue microarray BR1002a (US Biomax). Magnification used for all images is 400X **(A-C)** Tw1S4_AM6 was used to stain a breast cancer tissue microarray. The staining pattern of HSPG2 changes from interstitial to predominantly cellular with advancing stage, similar to that observed with Tw1S4_6.


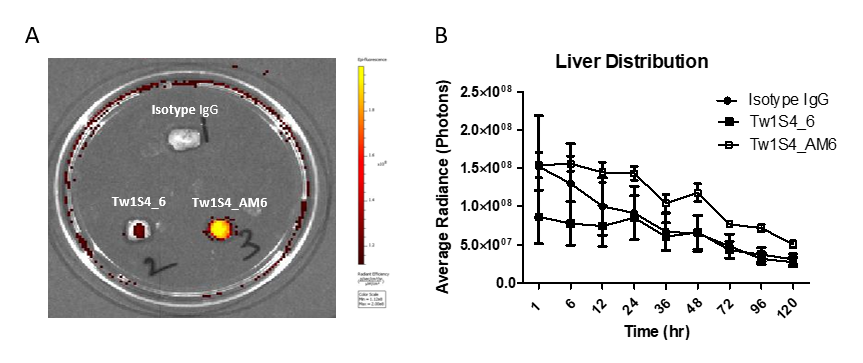


**Supplementary Figure 6:** Biodistribution studies for Tw1S4_6 and Tw1S4_AM6 **(A)** Tumors imaged *ex vivo* at 120 hours. One mouse from each group is shown in the image. **(B)** Quantified fluorescence values in liver. (P>0.05, two way ANOVA with multiple comparisons)


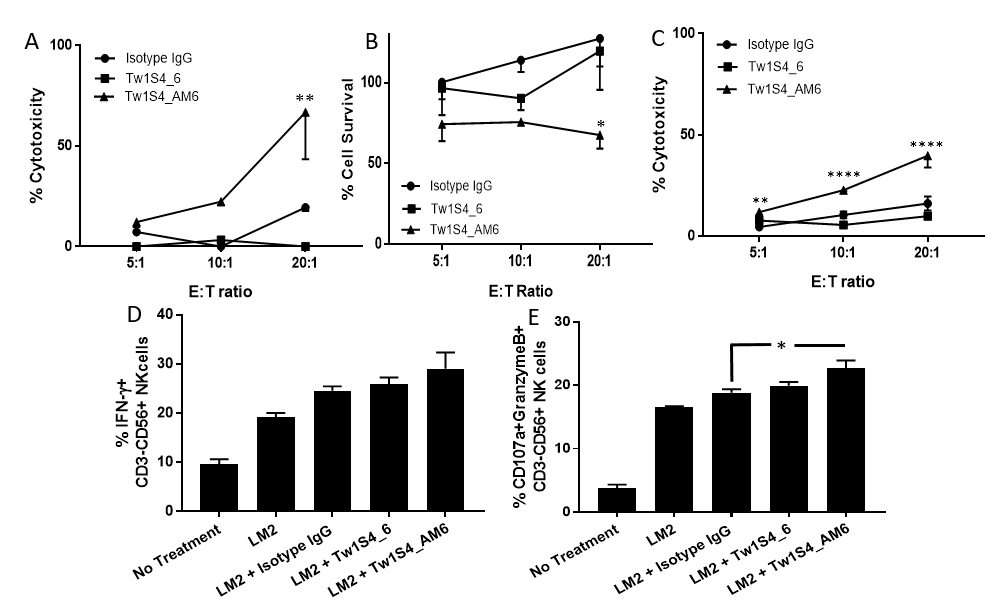


**Supplementary Figure 7:** *In vitro* ADCC assays with Tw1S4 antibodies **(A)** ADCC assay with human PBMCS from donor 1 showed significantly higher cytotoxicity with Tw1S4_AM6 (**P<0.01, two way ANOVA with Tukey’s multiple comparison tests, statistical significance is based off comparison between Isotype IgG and Tw1S4_AM6 at E:T 20:1) **(B)** and **(C)** ADCC assay with human PBMCS from donor 2 showed significantly higher cytotoxicity with Tw1S4_AM6 (**P<0.01, ****P<0.0001 two way ANOVA with Tukey’s multiple comparison tests, statistical significance is based off comparison between Isotype IgG and Tw1S4_AM6) **(D)** and **(E)** NK cell degranulation assays with human PBMCs from donor 2


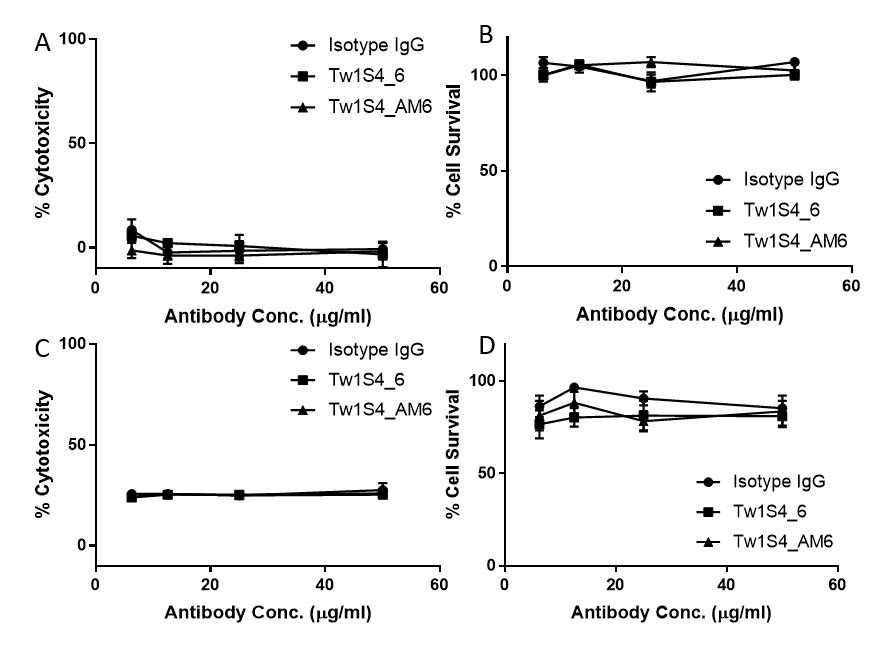


**Supplementary Figure 8:** *In vitro* CDC assays with Tw1S4 antibodies (A-B) CDC assay using human serum (C-D) CDC assay using mouse serum

**Supplementary Table 1:** A univariate cox regression to assess the overall association between HSPG2 expression and patient survival

|  | **coef** | **exp(coef)** | **se(coef)** | **z** | **p** |
| --- | --- | --- | --- | --- | --- |
| **expr** | 2.07 | 7.95 | 0.74 | 2.8 | 0.0052 |

**Supplementary Table 2:** A multi-variate cox regression to assess the overall association between HSPG2 expression and patient survival

|  | **coef** | **exp(coef)** | **se(coef)** | **z** | **p** |
| --- | --- | --- | --- | --- | --- |
| **expr** | 2.39 | 10.93 | 0.93 | 2.58 | 0.0098 |
| **age_at_diagnosis** | 0.082 | 1.086 | 0.023 | 3.64 | 0.00027 |
| **size** | -0.00244 | 1.00 | 0.020 | -0.12 | 0.91 |
| **grade** | 1.22 | 3.37 | 1.04 | 1.17 | 0.24 |
| **stage** | 0.74 | 2.09 | 0.41 | 1.80 | 0.071 |
| **lymph_nodes_pos** | 0.0013 | 1.00 | 0.062 | 0.02 | 0.98 |

**References**

1. Nesvizhskii, A. I., Keller, A., Kolker, E. & Aebersold, R. A statistical model for identifying proteins by tandem mass spectrometry. *Anal. Chem.* **75,** 4646–4658 (2003).

2. Decarlo, A. A. *et al.* Perlecan domain 1 recombinant proteoglycan augments BMP-2 activity and osteogenesis. *BMC Biotechnol.* **12,** 60 (2012).

3. Clackson, Tim. Lowman, H. B. Affinity Maturation of Phage Antibodies. in *Phage Display: A Practical Approach* (Oxford University Press, 2004).
